# Reporting a regular medical doctor index: A new measure of patient-physician affiliation for health administrative data

**DOI:** 10.1371/journal.pone.0314381

**Published:** 2024-12-02

**Authors:** Caroline King, M. Ruth Lavergne, Kimberlyn McGrail, Erin C. Strumpf, on behalf of the Quebec-British Columbia Patient Enrolment Project Team

**Affiliations:** 1 Department of Epidemiology, Biostatistics and Occupational Health, McGill University, Montreal, QC, Canada; 2 Department of Family Medicine, Dalhousie University, Halifax, NS, Canada; 3 Centre for Health Services and Policy Research, University of British Columbia, Vancouver, BC, Canada; 4 Department of Economics, McGill University, Montreal, QC, Canada; University of Calgary, CANADA

## Abstract

Having a regular medical doctor is associated with better process of care and health outcomes. The goal of this study was to harness the richness in health administrative data to create a measure which accurately predicted whether patients self-identified as having a regular medical doctor. The Canadian Community Health Survey (2007–2012) was linked with health administrative data (HAD) (2002–2012) from Quebec, Canada’s second largest province. The Canadian Community Health Survey includes respondents’ answer to whether they have a regular medical doctor, but health administrative data does not. We therefore used LASSO and Random Forests to build prediction models that predict whether a patient reports having a regular medical doctor using their data only available in the HAD. Our results show that predicting patient responses to ‘do you have a regular medical doctor?’ using an average of single-year Usual Provider Continuity over 3 years results in an area under the receiver operator characteristic curve of 0.782 (0.778–0.787). This was almost a 14% improvement in predictive accuracy compared to the frequently used single-year Usual Provider Continuity (0.688 (0.683–0.694)). We have called this new measure the Reporting a Regular Medical Doctor (RRMD) index. The RRMD index is easy to implement in HAD, is an elegant solution to the difficulties associated with low-users having unstable UPC scores, and brings a patient-oriented perspective to previous efforts to capture patient-physician affiliations in HAD. We recommend that researchers seeking to measure whether patients have a regular medical doctor using HAD consider using the RRMD index.

## Background

Having a regular medical doctor is associated with many processes of care and health outcomes including more preventative care, better management of chronic disease, increased satisfaction for patients and physicians, reduced emergency room usage and hospitalizations, and reduced costs [1–4]. It also contributes to better continuity of care and improved patient satisfaction [5]. The percentage of the population that has a regular medical doctor (RMD) is important because it serves as an indicator of access to primary care and can highlight inequities in access for specific populations [6–8]. Patients who do not have a regular medical doctor are more likely to use other services such as walk-in clinics or emergency departments which are viewed as a poor substitute for longitudinal primary care due to the interruption in coordination of care [4, 5, 9]. In Canada, despite more than a decade of primary care investments and reforms [10], the share of Canadians reporting that they have a regular medical doctor has remained stubbornly close to 85% from 2001 to 2022 [11, 12].

Patients’ report of having a RMD can be measured via survey questions such as “do you have a regular medical doctor?”. The respondent’s reasons for answering “yes” or “no” remain ambiguous, but likely include some combination of a trusting, caring relationship, repeated contacts with the same provider, and/or being formally enroled with a source of care. Insurers create administrative links between a patient and physician using formal rostering, enrolment, or empanelment. Patients’ affiliation to a primary care physician, that is, repeated contacts over time [7, 13–15], can be measured via concentration-of-care measures in health administrative databases. Concentration-of-care measures in health administrative data (HAD) are particularly popular because these data are routinely collected and therefore commonly used to evaluate the impacts of policy changes on access to and quality of care. In HAD, the most responsible physician for a patient’s care can be identified using attribution methods which assign patients to the provider with the highest percentage of services or total cost [16–18]. The Usual Provider Continuity (UPC) index [19], which captures the largest share of a patient’s visits with any one provider, is an example of a simple approach to identifying a patient’s RMD. These measures are associated with lower health care costs, lower hospitalization rates, fewer ED visits, higher immunization rates, and greater patient satisfaction [20–23]. Current HAD measures capture affiliation (repeated contacts over time), but they do not necessarily reflect the patient’s perspective: whether they consider themselves to have a regular medical doctor. The majority of the literature that attempts to identify patient-physician relationships in HAD [17, 18, 24–28] has focused on enumerating the physician’s patient roster (i.e., the perspective of the physician or the physician’s office manager). Of the few studies that focus on the patient’s perspective, only Shah et al. compared patient surveys to their indicators of affiliation [29]. They found high concordance between patient surveys and the largest share of visits to a family physician (FP) suggesting that predicting a patient’s RMD can be done successfully in administrative data; however, their study population was limited to people with diabetes and so the finding may not be generalizable to the general population.

Another limitation of most concentration of care measures is their sensitivity to utilization, where infrequent users cluster at certain values (i.e., 0, 0.5, 1). To avoid this issue, many researchers limit their data to persons with a minimum number of visits per year. However, this solution reduces the study population and limits the generalizability of the results.

HAD contains many variables which can be captured longitudinally, so it may be possible to better predict the patient perspective on what it means to have a RMD beyond simply repeated contacts. The primary goal of this paper was to create a model which predicts the patient’s response to the survey question “Do you have a regular medical doctor?” using only variables available in HAD. We then assessed similarities and differences between that new measure and traditionally used affiliation measures like UPC. We had a secondary goal of identifying which factors were most important for predicting having a RMD. The motivation for this was to understand the substantive contributors to patients’ responses to a simple yes/no question. The third objective was to create a simplified index using a small subset of the most important predictors. This measure will be useful for both researchers and government institutions where there is a lack of extensive data sources for monitoring rates of patient-physician affiliation over time and to evaluate the impacts of policy interventions on this health systems indicator.

## Methods

Using linked health survey and HAD, our general approach was to build a rich and extensive set of predictors using HAD and then use LASSO and Random Forests (RF) to build accurate predictive models. We then identified the most important predictors for each model and assessed if simple combinations of the most important predictors could be made into a single standardized score that could accurately predict self-reported responses to having a RMD. The McGill University Faculty of Medicine Institutional Review Board provided written approval after an expedited/delegated review (A05-E30-20A). No consent was obtained for this study: administrative and survey data were completely anonymized. The TorSaDE cohort includes Canadian Community Health Survey participants who provided consent to Statistics Canada for the linkage of their responses with data from other sources for research purposes.

### Data and study population

HAD data for Quebec 2002–2012 were linked to Statistics Canada’s Canadian Community Health Survey 2007–2012 (CCHS) by the TorSaDE working group [30]. Data was accessed between May 2020 and November 2020. HAD data in Quebec are collected by the provincial health ministry and the public insurer and include primary and specialty outpatient care, inpatient care, geographic, demographic and socioeconomic characteristics. It covers almost all Quebec residents with the exception of immigrants to the province in their first 3 months of residence, persons staying in Quebec temporarily, persons living on reserves and other Indigenous settlements in the province, and full-time members of the Canadian Forces [31]. There are a limited number of physicians (less than 15%) who are salaried in Quebec and the fee-for-service physician billing data do not capture visits to these physicians [32].

The CCHS is a nationally representative cross-sectional survey that collects information related to health status, health care utilization and health determinants for the Canadian population every two years [33] (similar to the Medical Expenditure Panel Survey in the US). The CCHS excludes: persons living on reserves and other Indigenous settlements in the provinces; full-time members of the Canadian Forces; the institutionalized population, children aged 12–17 that are living in foster care, and persons living in the remote Quebec health regions of Région du Nunavik and Région des Terres-Cries-de-la-Baie-James. Altogether, these exclusions represent less than 3% of the Canadian population aged 12 and over.

We have a total of 61,083 individual responses to the question do you have a regular medical doctor in Quebec from 3 cycles of the CCHS (2007, 2009, 2012). We limit our use to patients that respond ‘yes’ or ‘no’ because there were only 114 respondents who said ‘don’t know’ or did not answer. CCHS data for each respondent are linked with their HAD data from 1996 until 2016 for all respondents that gave permission to have their data linked (92.8% of Quebec CCHS participants) [34]. This is the outcome of interest for our predictive modelling. Because we do not have information on which particular physician a respondent considered their regular medical doctor, we do not exclude respondents whose family physician changed over the study period.

We consider two study populations: the full linked CCHS population (N = 60,968) and a subpopulation of respondents who had at least 1 ambulatory visit every year in the 5 years prior to the survey (N = 35,184). We refer to this cohort as ‘regular users’. We included the regular users cohort because we hypothesised that measures of affiliation would be more accurate among persons in this group.

### Building predictors

To take full advantage of the data available, we created many relevant indicators to include in our prediction models. We identified indicators used to represent the patient-physician relationship in administrative data based on the literature and also used stakeholder input through in-person meetings with clinicians and patient partners during which they identified factors they associated with having a RMD. For example, one suggestion we could measure with HAD was having weekend access to their family doctor.

We categorized all 74 predictors into 3 groups: 1) Demographics, 2) Health Status and 3) Health Services Utilization. Briefly, we created variables for age, sex, rurality, neighbourhood level socio-economic status, indices for the presence of specific chronic diseases (e.g., COPD, diabetes, mental health and coronary heart disease) and measures of comorbidity (Charlson comorbidity index), counts and averages of types of visits (FP, specialists, ER, weekend and hospitalizations) and ten indicators of affiliation that reflect the concentration of provider contacts (e.g., Usual Provider Continuity, Modified Continuity Index, and Wolinsky’s Continuity of care; see S1 Table for the full list [19, 35–38]). We created all variables for the year before the survey and counts and averages for all of the visit types, chronic disease indicators, and affiliation measures over 3- and 5-years prior. The 3- and 5-year affiliation measures were made by averaging the measures calculated in single years. For full lists of created indicators, how they were constructed and references, see S1 Table.

### Predictive models and variable importance

We used Random Forest (RF), a machine learning technique, and LASSO to build our predictive models for having a RMD. We used two approaches to build our prediction models because they have different strengths and weaknesses and so comparing results from both approaches allows for a richer interpretation [39]. We compared the model performances in terms of Area Under the Receiver Operator Curve (AUC). We compared these AUC estimates to a third logistic regression (LR) model of the standard 1-year UPC index on having a RMD.

### Random Forests

Briefly, RF is a non-parametric method used to predict an outcome by building hundreds of decision trees through bootstrapping. Decision trees are flow-chart like structures that subset the data over and over again until the outcome is the same, or close to, for everyone in the final subsets. Algorithms for constructing decision trees work top-down, by choosing a variable at each step that is best able to subset the data into homogeneous groups in terms of the outcome [40, 41]. The final RF model is a ‘black box’ solution that is based on the average tree. We used Minimal Depth variable selection which selects variables based on their position in the tree [42, 43]. The variables that are used early in the tree to subset the data are kept in the model.

RF does not produce beta coefficient estimates and instead uses measures of variable importance (VIMP) to evaluate the relationships between the outcome and predictors. VIMP is assessed by measuring changes in prediction accuracy when the values of a variable are randomly shuffled (permuted) while all other variables are constant [40]. This process keeps the variable in the model but effectively removes its correlation with the outcome.

Rare or very common outcomes create difficulties for RF because its decisions are based on classification error and a low error rate can be achieved by simply classifying all the observation as members of the majority class [44]. To overcome this challenge, we built each tree on a sample which intentionally under-samples from the outcome class that is most common (responding “yes” to having a RMD) [45]. Prediction accuracy is assessed by testing each tree in the forest on the data that was left out of its bootstrap sample. This is referred to as the Out-of-Bag error and provides an unbiased, or slightly conservative estimate of the true error rate of the model [40, 46, 47]. We used the R package randomForestSRC to build our RF models [42]. Additional detail on how RF was used in this analysis can be found in [39].

### LASSO

LASSO (least absolute shrinkage and selection operator) is a regression analysis method that does both variable selection and regularization by adding a penalty equal to theabsolute value of the coefficients. This simplifies the model by forcing certain coefficients to be set to zero [48]. We chose LASSO because shrinkage methods are useful when the focus is on obtaining accurate predictions [49–51]. To measure variable importance with LASSO, we used the standardized beta weights from the final regression model. We chose lambda (the magnitude of the penalty) using cross-validation which is preferable when prediction is the goal. We also used 5-fold cross-validation to assess the prediction accuracy of our model measured using AUC. We used the Stata lasso package for our analysis.

### Correlated variables in RF and LASSO

The result of creating an extensive set of predictors is that many are highly correlated. For example, the number of visits a person has with FPs and the number of all ambulatory visits in a year. Both RF and LASSO perform well in the presence of highly correlated predictors [52, 53], but they handle them in different ways which impacts the VIMP ratings and ultimately our interpretations. In general, if there are two highly correlated features in the data, RF will distribute the VIMP of two correlated variables evenly between both variables [54, 55]. In contrast, LASSO will select one and remove the other, but this does not mean the other is not important [53].

### Making a simple measure: Standardized scores

We had 106 predictors available for our models to choose from, but since not all researchers will have access to such rich data, or wish to create all these predictors, we created a second set of models with reduced dimensionality. Using the VIMP rankings from both the RF and LASSO models, we selected the most important predictors for reporting having a RMD. We examined the prediction performance of each high-ranking variable through 5-fold cross-validation to get AUC estimates. We also standardized the variables and added them together in several combinations to create simple scores and test their predictive accuracy. Confidence intervals for all AUC estimates were calculated using bootstrapping with 500 repetitions. We create a new measure which we call Reporting a Regular Medical Doctor (RRMD) based on our results.

## Results

**Fig 1:**
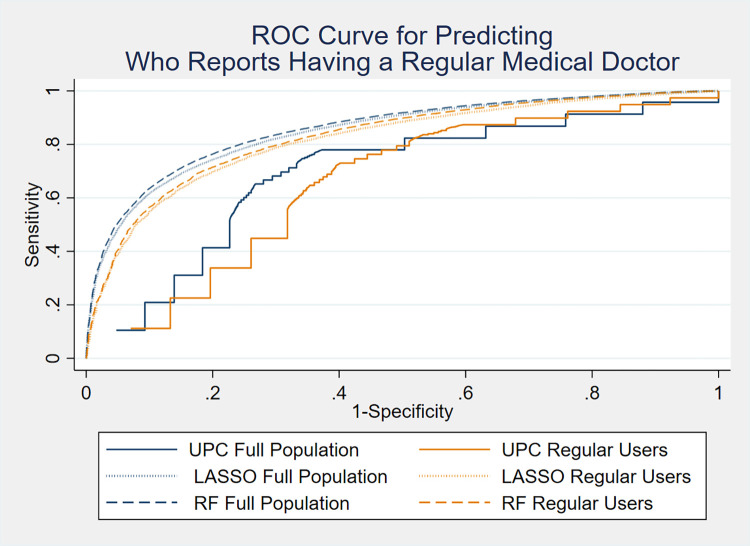
Receiver operating characteristic (ROC) curves for the RF and LASSO models, and the UPC index. Curves are shown for both the full cohort and the regular users.

Fig 1 shows the receiver operating characteristic (ROC) curves for the RF and LASSO models and the UPC index. Their corresponding AUC estimates are in S2 Table. An AUC of 0.5 indicates the model is no better than chance at predicting the outcome and 1 indicates a perfect predictor. Here we will consider AUC scores of 0.5–0.7 as poor, 0.7 to 0.8 as acceptable, 0.8 to 0.9 as excellent, and more than 0.9 as outstanding. These cut-offs are arbitrary but helpful for interpreting results and commonly used [56]. The performance of each model is shown for the full population and regular users. The models consistently perform better on the full population (blue lines) than regular users (orange lines). RF and LASSO perform similarly with excellent AUC scores of 0.86 and 0.85 respectively. The AUC for UPC is substantially lower and performs poorly with an AUC score of 0.69.

### VIMP graphs

**Fig 2:**
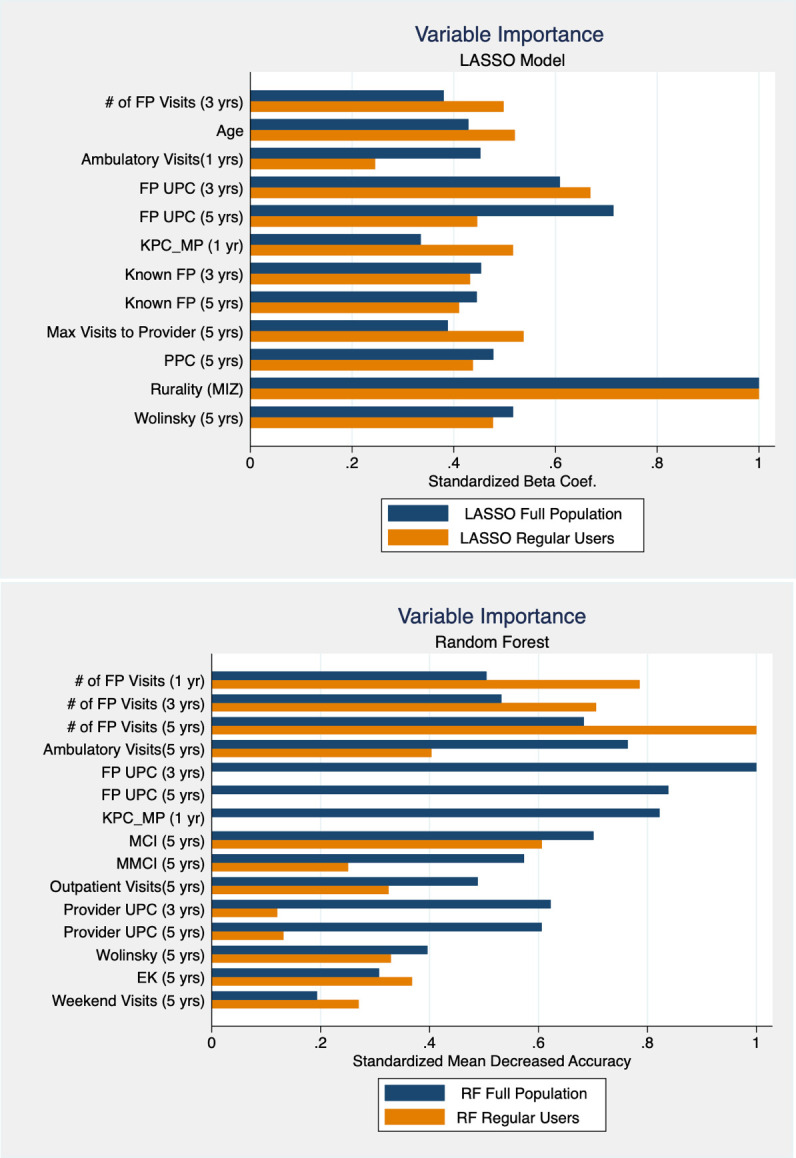
Variable importance scores for LASSO and Random Forest models. For each model, the VIMPs are divided by the maximum VIMP value to scale them so that the most important variable is equal to 1. FP_UPC: Usual provider continuity limited to family physician visits. KPC_MP: Known Provider Continuity-Multiple Providers. MCI: Modified Continuity Index. MMCI: Modified, Modified Continuity Index. Provider UPC: Usual provider continuity based on all provider visits. EK: Ejlertsson’s Index K. Known FP: Known Provider Continuity -Personal Provider. PPC: Personal Provider Continuity.

The predictors with the top VIMP scores, in alphabetical order, are shown for the LASSO and RF models in Fig 2A and 2B, respectively. Variables were included if they ranked within the top ten predictors for models based on the full population or on regular users. The models have differences, but both clearly emphasize predictors related to FP utilization, such as number of visits and UPC, as the most important predictors. The 5- and 3-year averages of single year UPC clearly dominate the 1-year averages suggesting that longer term utilization patterns are better predictors. Rurality is an anomaly in that it ranked as most important in the LASSO models but ranked much lower in the RF (standardized mean decreased accuracy: full population = 0.036; regular users = 0.047). All of our indicators of health status, such as the indicator for diabetes or the Charlson comorbidity index, had very low VIMP scores across all models and are not shown in Fig 2.

Restricting to regular users led to small changes in the VIMP rankings for the LASSO models but substantial changes in the RF, in part because RF is very sensitive to changes in the prevalence of the outcome. The AUC estimates, from Fig 1 and S2 Table, show that despite the changes in the RF variable rankings between the two populations, the model still performs well; it just relies on a different set of variables.

We selected the highest-ranking variables based on VIMP results and assessed their ability to predict reporting having a RMD (Table 1). UPC, number of FP visits and Known Provider Continuity—Multiple Providers (KPC_MP) perform well when using longer term averages (i.e., 3 and 5-years), producing AUC estimates between 0.75 and 0.8 (scores 2, 3, 8 and 9). These AUC estimates are only slightly less than the full RF and LASSO model estimates which are approximately 0.85 (scores 17 and 18).

**Table 1:** AUC estimates for selected models with a limited set of predictive variables relative to full RF and LASSO models.

Score	Variables Included in Score	AUC Full Population	Regular Users	Total Number of Variables
*1*	UPC 1-year	0.688 (0.683–0.694)	0.651 (0.639–0.663)	1
*2*	UPC 3-year	0.782 (0.778–0.787)	0.731 (0.722–0.742)	1
*3*	UPC 5-year	0.798 (0.794–0.803)	0.741 (0.733–0.750)	1
*4*	FP Visits 1-year	0.702 (0.697–0.707)	0.642 (0.632–0.651)	1
*5*	FP Visits 3-year	0.750 (0.747–0.755)	0.650 (0.639–0.661)	1
*6*	FP Visits 5-year	0.759 (0.755–0.764)	0.646 (0.636–0.655)	1
*7*	KPC_MP 1-year	0.715 (0.711–0.720)	0.679 (0.669–0.690)	1
*8*	KPC_MP 3-year	0.775 (0.772–0.780)	0.712 (0.702–0.722)	
*9*	KPC_MP 5-year	0.792 (0.788–0.797)	0.716 (0.706–0.726)	1
*10*	Rurality	0.537 (0.531–0.543)	0.544 (0.534–0.554)	1
*11*	Age	0.688 (0.683–0.693)	0.691 (0.682–0.701)	1
*13*	UPC 3-year + FP Visits 3-year	0.799 (0.795–0.804)	0.747 (0.737–0.756)	2
*14*	UPC 3-year + FP Visits 3-year + KPC_MP 3-year	0.821 (0.817–0.825)	0.783 (0.775–0.791)	3
*15*	UPC 3-year + FP Visits 3-year + KPC_MP 3-year + Rurality	0.820 (0.816–0.824)	0.772 (0.763–0.780)	4
*16*	UPC 3-year + FP Visits 3-year + KPC_MP 3-year + Rurality + Age	0.824 (0.820–0.827)	0.787 (0.779–0.795)	5
*17*	Full RF Model	0.858 (0.854–0.861)	0.829 (0.821–0.835)	69
*18*	Full LASSO Model	0.848 (0.845–0.852)	0.812 (0.810–0.825)	55

Standardizing the variables and then adding them together allowed us to create aggregate scores or indices which incorporate information from several of the top-ranking variables. These simple combinations of variables achieve excellent predictive power, though still slightly lower than the full LASSO and RF models. Combining a few utilization measures, like the 3-year averages of UPC, FP visits and KPC_MP (score 14), leads to a small improvement over their individual AUC scores (scores 2, 5 and 8 respectively). However, adding in rurality and age (score 15 and 16) contribute little to improving the AUC estimate.

It is clear from Table 1 that using a single measure of FP utilization averaged over 3 or 5 years of single year measures can predict reporting having a RMD acceptably well. Table 1 is far from an exhaustive list of variable combinations, the combinations included demonstrate that adding additional variables has diminishing returns. Based on these results, we propose a new HAD measure of affiliation that uses a 3-year average of UPC. We call this new measure Reporting a Regular Medical Doctor Index or RRMD index. While the 5-year average of UPC performs better than 3-years, the additional 2 years of data could be restrictive. This is a patient-oriented measure because it predicts patients’ report of having a RMD. Although we recommend using RRMD index in its continuous form, we provide the overall error, sensitivity and specificity for a range of cut-points (S3 Table).

Table 1 AUC estimates for the most important variables for predicting having a regular medical doctor, based on variable importance scores from the LASSO and Random forest models. For the scores with more than one variable (scores 13 and above), each variable was standardized and the aggregate score of the variables was used to predict having a regular medical doctor.

## Discussion

The first goal of this work was to harness the richness in HAD data to create a measure which predicted the patient’s survey-based reports of having a RMD. We were able to make significant improvements over existing HAD affiliation measures in predicting RRMD. Our most accurate model, the full RF, was almost 25% better than the 1-year UPC in terms of AUC (0.17 absolute difference). Contrary to our hypothesis, all the models performed better when using the full cohort as opposed to the regular users cohort. We believe this is because limiting to only regular users removes a significant proportion of the population that reports not having a regular medical doctor (S4 Table).

The second objective was to identify which factors were most important for predicting reporting having a RMD. Our results overwhelmingly point to averages of single-year FP utilization measures over longer time periods (UPC, KPC_MP and FP visits) as the most important predictors available in HAD of patients’ self-report of having a RMD. This remains the case even when we restrict our cohort to regular healthcare users. Due to documented correlations between age, sex, and health status with reporting having a RMD [2, 3, 57, 58], we had anticipated these predictors would increase in importance when the cohort was restricted to healthcare users, but this was not the case. Restricting the cohort led to very similar VIMP results for the LASSO model which still highlighted FP utilization averages over longer time periods and rurality as top predictors. In RF, restricting the cohort led to changes, but only among variables within the health care utilization category (i.e., priority changed from UPC to FP visits). The results regarding the importance of the rurality measure are inconclusive given its high ranking in the LASSO model and very low ranking in the RF model. However, its predictive ability (Table 1, score 10) is hardly better than chance in our context. Urban/ rural differences in access to primary care have been found in other studies [2, 59], but the relationship is likely dependent on physician supply which can be highly contextualized [60].

Our third objective was to create a new patient-centered measure of affiliation for HAD. Based on our results, we suggest using a 3-year average of single year UPC which led to approximately 14% improvement over 1-year UPC in predicting having a RMD. We call our new measure the Reporting a Regular Medical Doctor Index or RRMD index. The more complex models provide modest improvements in predictive accuracy over the RRMD index; but there is a trade-off between these modest gains and logistical considerations (i.e., data availability and analyst’s time to generate indicators). For instance, the 5-year UPC average performs slightly better than the 3-year UPC average but requiring 5-years of data may not always be practical. Adding FP visits to UPC (score 13) has a small advantage over using 3-year UPC, but this requires the additional steps of standardizing and combining the two indicators. Furthermore, adding demographic variables to the measure may introduce some modelling complications if researchers wish to stratify or adjust for those demographics. Ultimately, researchers can choose the measure in Table 1 that best suits their needs; however, we recommend a single measure, which can be easily applied in a variety of settings, to promote the use of a standard measure that can be compared across studies.

There is a clear explanation for the considerable improvement seen in the AUC estimates by using UPC averaged over 3 or 5-years instead of 1: the changes in distribution of UPC scores. UPC scores are sensitive to utilization levels, where infrequent users cluster at certain UPC values (i.e., 0, 0.5, 1). This characteristic is well known and seen as a major limitation of the UPC index [61]. Examining utilization patterns over longer periods smooths the distribution of UPC scores which then allows for a better prediction of reporting having a RMD. It could also be the case that patients consider their longer-term utilization patterns when considering their response to the survey question.

Our results are consistent with previous work which found that FP visits could be used to predict a patient’s RMD within a diabetic cohort [29]. Other work comparing patients with a RMD vs those who do not has also found associations with age, sex [2, 3, 7, 58, 62] and health status in terms of the number of chronic conditions [3, 7, 58]. While our descriptive statistics (S4 Table) also demonstrate these associations, the prediction models were dominated by the healthcare utilization measures in both the LASSO and RF. The likely reason for this is that comorbidities, sex and age are all highly correlated with healthcare utilization (i.e., the utilization measures are already capturing this information indirectly).

Many health systems are moving towards interprofessional team-based models of primary care (e.g., primary care medical homes). These transitions are shifting the degree to which patient care is concentrated with a single physician with patients moving from having a RMD to having a usual place of care [9]. This could disrupt the relationships between patients and their physicians that are central to primary health care. The RRMD indicator could be useful for understanding how these changes are affecting the proportion of patients reporting having a RMD over time.

While responses to having an RMD has been used for decades as a performance measure in Canada and other countries (for example, the US National Health and Nutrition Examination Survey, the Health Survey for England, the Australian Health Survey, and the Commonwealth Fund International Health Policy Survey), there remains ambiguity regarding what patients are thinking when they respond to this question, and reasons for responses likely vary by patient. Reporting having a RMD is clearly associated with FP utilization, but there are likely many other factors such as registration/ enrolment with a physician or trusting the physician which would contribute to a patient’s response that we cannot capture. Having data on these additional factors could improve the predictive accuracy of our models. Our measure is also limited in that it predicts whether a person reports having a RMD—not who that doctor is, nor the other providers in the same clinic. Another limitation of our work relates to the representativeness of our sample. The CCHS uses sample weights to make the cohort representative of the Canadian population. We did not adjust for the weights because of the difficulties it presents in our models. We include the means and standard errors for the measured characteristics in our cohort for both the weighted and unweighted samples (S5 Table) for comparative purposes which are similar, but small differences exist. Because fee-for-service payment remains dominant in Canadian primary care, our findings may not generalize to health care systems where family physicians and/or patients face financial incentives to maintain affiliation.

Our results demonstrate that the abundance of available data in HAD is underutilized when measuring having a RMD. Our RF and LASSO models take full advantage of the available data and perform substantially better than 1-year UPC at predicting having a RMD. What is more notable is how well simply using 3 -year averages of UPC, instead of 1-year, improves the prediction of reporting having a RMD. While this may appear to be an obvious outcome, it has never been suggested as a solution for improving predictive accuracy of UPC or addressing the problematic UPC sensitivity to utilization. We recommend that researchers seeking to measure or adjust for which patients report having a RMD consider using the RRMD index. The RRMD index is easy to implement in HAD, is an elegant solution to the difficulties associated with low-users having unstable UPC scores and brings a patient-oriented perspective to previous efforts to predict patient-physician affiliations in HAD.

## Supporting information

S1 Table List of variables. (DOCX)

S2 Table Area Under the Receiver Operator Curve (AUC) estimates for the UPC index, LASSO, and Random Forest (RF) models. (DOCX)

S3 Table Prediction performance at various binary cut-points for Reporting a Regular Medical Doctor with 3- and 5-years of data. A cut-point of 0.10 indicates that anyone with a predicted probability of having a regular medical doctor greater than or equal to 0.1 is classified as having a regular medical doctor and anyone with less than 0.1 is classified as not having a regular medical doctor.
(DOCX)

S4 Table Characteristics of Canadian Community Health Survey (CCHS) respondents reporting having a regular medical doctor and those who reported not having a regular medical doctor. Characteristics are reported for the full CCHS cohort and for a cohort restricted to those that had at least one ambulatory visit in each year in the 5-years prior to the survey (regular users).
(DOCX)

S5 Table Characteristics of Canadian Community Health Survey (CCHS) respondents reporting having a regular medical doctor and those who reported not having a regular medical doctor. Characteristics are reported for the full unweighted and weighted CCHS cohort.
(DOCX)
