# Supplementary material for: Reporting a regular medical doctor index: A new measure of patient-physician affiliation for health administrative data
Source: PLoS One. 2024 Dec 2;19(12):e0314381. doi: 10.1371/journal.pone.0314381 (PMC11611086; doi:10.1371/journal.pone.0314381)
Supplement: S1 Table — (DOCX) [file pone.0314381.s001.docx]

**S1 Table. List of Variables**

| Category | Variable | Description/ formula | Source |
| --- | --- | --- | --- |
| Demographics | Age | Continuous indicator: age of person at time of survey |  |
|  | Sex | Binary indicator of sex |  |
|  | SES | Ordinal indicator with (5 levels): area code based socioeconomic measure of neighbourhood income per person equivalent, adjusted for household size, which is released by Statistics Canada (QIAPPE). | Statistics Canada |
|  | Rurality | Categorical indicator (7 levels): area code-based measure of rurality developed by Statistics Canada (CSIZEMIZ). | Statistics Canada |
| Healthcare Utilization | Ambulatory visits | Continuous indicator: Count of all outpatient visits not including ER visits. |  |
|  | Outpatient Visits | Continuous indicator: Count of all outpatient visits. |  |
|  | ER visits | Continuous indicator: Count of all ER visits |  |
|  | Weekend Visits | Continuous indicator: Count of visits that occurred on a weekend. |  |
|  | Usual Provider Continuity | Continuous indicator (0-1): The fraction of visits to the most frequently visited provider. | Breslau N, Reeb KG. Continuity of care in a university-based practice. *J Med Educ*. 1975;50(10):965-969. |
|  | Usual Provider Continuity: Binary | Binary indicator: 1 if the fraction of visits to the most frequently visited provider is >0.75; otherwise 0 | Menec VH, Sirski M, Attawar D. Does Continuity of Care Matter in a Universally Insured Population? *Health Serv Res*. 2005;40(2):389. |
|  | Usual Provider Continuity limited to FP visits | See Usual Provider Continuity |  |
|  | Usual Provider Continuity Binary limited to FP visits | See Usual Provider Continuity Binary |  |
|  | Wolinsky | Binary indicator: 1 if there was at least 1 visit to the same provider every 8 months over the previous 2-year period. | Wolinsky FD, Miller TR, Geweke JF, et al. An interpersonal continuity of care measure for medicare part B claims analyses. *Journals Gerontol - Ser B Psychol Sci Soc Sci*. 2007;62(3). |
|  | Modified Continuity Index | Continuous indicator: Equals 1 minus the number of providers divided by the number of visits. This index is adjusted for utilization by ascribing a higher value to those who have more frequent visits to the same provider. | Sturmberg JP, Schattner P. Personal doctoring. Its impact on continuity of care as measured by the comprehensiveness of care score. *Aust Fam Physician*. 2001;30(5):513-518. |
|  | Personal Provider Continuity | Binary indicator: A dichotomous version of the Modified Continuity index. | Sturmberg JP, Schattner P. Personal doctoring. Its impact on continuity of care as measured by the comprehensiveness of care score. *Aust Fam Physician*. 2001;30(5):513-518. |
|  | Modified, Modified Continuity Index | Continuous indicator: Modified Continuity Index divided by 1 minus the inverse number of visits. | Magill M, Senf J. A new method for measuring continuity of care in family practice residencies. *J Fam Pract*. 1987 |
|  | Ejlertsson’s Index K | Continuous indicator:  (total # visits - # providers) / (total visits-1) | Ejlertsson Gör, Berg S. Continuity of Care in Health Care Teams a Comparison of Continuity Measures and Organisational Solutions. *Scand J Prim Health Care*. 1985;3(2):79-85. |
|  | Number of providers seen | Continuous indicator: Total number of providers seen in year |  |
|  | Number of specialists seen | Continuous indicator: Total number of specialists seen in year |  |
|  | Known provider of care (Multiple providers) | Continuous indicator:  Number of visits in year t with the physicians seen in the year t-1 /Total # of visits in year t | Tousignant P, Diop M, Fournier M, et al. Validation of 2 new measures of continuity of care based on year-to-year follow-up with known providers of health care. *Ann Fam Med*. 2014;12(6):559-567. |
| Health Status | Charlson Index | Comorbidity index |  |
|  | Diabetes  ICD-9: 250 excl: 650-659 (pregnancy)  ICD-10: E10-E14 | Binary indicator: 1 if 1 hospitalization or 2 outpatient visits within 2 years; otherwise 0 | Canadian Chronic Disease Surveillance System (CCDSS) |
|  | COPD  ICD-9: 491- 492; 496  ICD-10: J41-J44 | Binary indicator: 1 if 1 hospitalization or 1 outpatient visits within 1 year; otherwise 0 | Canadian Chronic Disease Surveillance System (CCDSS) |
|  | Coronary Artery Disease (CHD)  ICD-9: 410-414  ICD-10: I20-I25 | Binary indicator: 1 if hospitalization or 2 outpatient visits within 2 years; otherwise 0 | Canadian Chronic Disease Surveillance System (CCDSS) |
|  | Mental Health Conditions  ICD-9: 295- 302; 306-319  ICD-10: F20-F54; F56-F99 | Binary indicator: 1 if 1 hospitalization or 2 ambulatory visits within 1 year; otherwise 0 | Canadian Chronic Disease Surveillance System (CCDSS)  We deviate from the CCDSS in that we do not include substance abuse in our definition because we looked at it separately (291, 292, 303, 304, 305: F10 – F18, F55). |
