# Supplementary material for: Reporting a regular medical doctor index: A new measure of patient-physician affiliation for health administrative data
Source: PLoS One. 2024 Dec 2;19(12):e0314381. doi: 10.1371/journal.pone.0314381 (PMC11611086; doi:10.1371/journal.pone.0314381)
Supplement: S2 Table — (DOCX) [file pone.0314381.s002.docx]

**S2 Table. Area Under the Receiver Operator Curve (AUC) estimates for the UPC index, LASSO, and Random Forest (RF) models**

| **Model** | **AUC** |
| --- | --- |
| UPC Full Population | .688 (.683 - .694) |
| UPC At Least 1 Visit | .651 (.639 - .663) |
| LASSO Full Population | .848 (.845 - .852) |
| LASSO At Least 1 Visit | .812 (.810 - .825) |
| RF Full Population | .858 (.854 - .861) |
| RF At Least 1 Visit | .829 (.821 - .835) |
