# Supplementary material for: Reporting a regular medical doctor index: A new measure of patient-physician affiliation for health administrative data
Source: PLoS One. 2024 Dec 2;19(12):e0314381. doi: 10.1371/journal.pone.0314381 (PMC11611086; doi:10.1371/journal.pone.0314381)
Supplement: S3 Table — A cut-point of 0.10 indicates that anyone with a predicted probability of having a regular medical doctor greater than or equal to 0.1 is classified as having a regular medical doctor and anyone with less than 0.1 is classified as not having a regular medical doctor. (DOCX) [file pone.0314381.s003.docx]

**S3 Table. Prediction performance at various binary cut-points for Reporting a Regular Medical Doctor with 3- and 5-years of data**. A cut-point of 0.10 indicates that anyone with a predicted probability of having a regular medical doctor greater than or equal to 0.1 is classified as having a regular medical doctor and anyone with less than 0.1 is classified as not having a regular medical doctor.

| RRMD Number of years used | Cut-Point | Overall Error | Sensitivity | Specificity |
| --- | --- | --- | --- | --- |
| 3 year | 0.90 | 19.6% | 91.5% | 39.6% |
|  | 0.80 | 20.0% | 89.8% | 44.1% |
|  | 0.70 | 20.5% | 88.5% | 46.5% |
|  | 0.60 | 24.7% | 77.4% | 67.7% |
|  | 0.50 | 28.2% | 70.9% | 75.0% |
|  | 0.40 | 31.5% | 65.8% | 78.1% |
|  | 0.30 | 43.0% | 47.9% | 90.6% |
|  | 0.20 | 48.1% | 40.7% | 92.9% |
|  | 0.10 | 58.2% | 26.9% | 96.2% |
| 5 year |  |  |  |  |
|  | 0.90 | 19.5% | 94.7% | 28.4% |
|  | 0.80 | 19.6% | 93.4% | 32.6% |
|  | 0.70 | 20.5% | 87.1% | 51.6% |
|  | 0.60 | 22.1% | 83.1% | 58.8% |
|  | 0.50 | 27.7% | 71.3% | 75.8% |
|  | 0.40 | 31.7% | 65.1% | 80.3% |
|  | 0.30 | 42.4% | 48.3% | 91.5% |
|  | 0.20 | 49.6% | 38.4% | 94.2% |
|  | 0.10 | 64.0% | 18.9% | 98.3% |
