# Supplementary material for: Reporting a regular medical doctor index: A new measure of patient-physician affiliation for health administrative data
Source: PLoS One. 2024 Dec 2;19(12):e0314381. doi: 10.1371/journal.pone.0314381 (PMC11611086; doi:10.1371/journal.pone.0314381)
Supplement: S4 Table — Characteristics are reported for the full CCHS cohort and for a cohort restricted to those that had at least one ambulatory visit in each year in the 5-years prior to the survey (regular users). (DOCX) [file pone.0314381.s004.docx]

**S4 Table. Characteristics of Canadian Community Health Survey (CCHS) respondents reporting having a regular medical doctor and those who reported not having a regular medical doctor.** Characteristics are reported for the full CCHS cohort and for a cohort restricted to those that had at least one ambulatory visit in each year in the 5-years prior to the survey (regular users).

|  | Total for full CCHS cohort | Report not having a regular medical doctor | Report having a regular medical doctor | Total for Regular Users Cohort | Report not having a regular medical doctor | Report having a regular medical doctor |
| --- | --- | --- | --- | --- | --- | --- |
|  | N=60,968 | N=13,077 | N=47,891 | N=35,184 | N=3,777 | N=31,060 |
| Age Group N (%) |  |  |  |  |  |  |
| 12-19 years old | 10.91% | 15.72% | 9.59% | 5.91% | 10.81% | 5.40% |
| 20-29 years old | 12.53% | 22.44% | 9.79% | 8.72% | 21.21% | 7.43% |
| 30-39 years old | 13.68% | 21.20% | 11.61% | 10.54% | 18.87% | 9.67% |
| 40-49 years old | 12.82% | 15.53% | 12.07% | 10.38% | 12.80% | 10.13% |
| 50-59 years old | 18.10% | 13.88% | 19.26% | 19.42% | 15.76% | 19.79% |
| 60-69 years old | 16.77% | 7.31% | 19.38% | 22.10% | 11.83% | 23.16% |
| 70-79 years old | 10.21% | 2.87% | 12.23% | 15.12% | 5.64% | 16.10% |
| 80+ years old | 4.98% | 1.04% | 6.06% | 7.81% | 3.08% | 8.30% |
|  |  |  |  |  |  |  |
| Female N(%) | 54.05% | 39.50% | 58.06% | 63.88% | 60.26% | 64.25% |
| Neighbourhood Income Quintile N(%) |  |  |  |  |  |  |
| Lowest | 19.75% | 20.77% | 19.47% | 19.74% | 20.34% | 19.67% |
| 2 | 19.70% | 20.68% | 19.44% | 19.55% | 20.52% | 19.45% |
| 3 | 19.86% | 19.22% | 20.04% | 19.76% | 18.75% | 19.86% |
| 4 | 19.87% | 19.55% | 19.96% | 19.87% | 20.15% | 19.84% |
| Highest | 20.72% | 19.67% | 21.00% | 21.01% | 20.15% | 21.09% |
| Rurality (Census SizeMIZ)*  N(%) |  |  |  |  |  |  |
| 1,500,000 + | 29.93% | 37.78% | 27.77% | 29.85% | 40.49% | 28.75% |
| 500,000 –1,499,999 | 12.90% | 11.85% | 13.18% | 13.13% | 11.62% | 13.29% |
| 100,000 – 499,999 | 10.20% | 10.00% | 10.25% | 10.40% | 9.25% | 10.52% |
| 10,000 – 99,999 (any CMACA < 100,000) | 17.02% | 15.54% | 17.43% | 17.36% | 16.10% | 17.50% |
| Non-CMACA; Strong MIZ | 5.59% | 4.74% | 5.83% | 5.46% | 3.64% | 5.65% |
| Non-CMACA; Moderate MIZ | 12.41% | 9.17% | 13.30% | 12.68% | 8.84% | 13.08% |
| Non-CMACA; Weak / No MIZ | 11.95% | 10.92% | 12.23% | 11.11% | 10.06% | 11.22% |
| Mean # of Ambulatory visits 1 yr (std) | 5.34 (9.17) | 2.25 (5.44) | 6.20 (9.78) | 7.51 (10.83) | 4.82 (8.54) | 7.79 (11.00) |
| Mean # of Ambulatory visits 2 yr (std | 5.18 (7.05) | 2.22 (4.20) | 6.00 (7.45) | 7.48 (8.15) | 5.17 (6.54) | 7.72 (8.26) |
| Mean # of Ambulatory visits 3 yr (std | 5.08 (6.30) | 2.24 (3.87) | 5.87 (6.60) | 7.44 (7.16) | 5.43 (6.06) | 7.64 (7.23) |
| Mean # of FP visits 1 yr (std) | 2.98 (4.63) | 1.33 (2.58) | 3.44 (4.95) | 4.05 (5.38) | 2.50 (3.71) | 4.21 (5.50) |
| Mean # of FP visits 3 yrs (std) | 2.96 (3.57) | 1.34 (1.97) | 3.41 (3.78) | 4.14 (4.06) | 2.79 (2.82) | 4.28 (4.15) |
| Mean # of FP visits 5 yrs (std) | 2.94 (3.21) | 1.36 (1.83) | 3.38 (3.37) | 4.17 (3.59) | 2.94 (2.61) | 4.30 (3.65) |
| Mean # of ED visits (std) | 0.59 (1.59) | 0.46 (1.31) | 0.63 (1.66) | 0.75 (1.83) | 0.77 (1.74) | 0.75 (1.84) |
| Mean # of Weekend visits (std) | 0.42 (1.72) | 0.26 (0.98) | 0.47 (1.87) | 0.57 (2.11) | 0.46 (1.44) | 0.58 (2.17) |
| UPC Index Mean (std) | 0.49 (0.50) | 0.24 (0.42) | 0.55 (0.50) | 0.58 (0.49) | 0.34 (0.47) | 0.61 (0.49) |
| Known Provider Continuity 1 yr (std) | 59.47 (38.04) | 38.32 (40.45) | 65.30 (35.17) | 73.84 (27.33) | 64.60 (29.29) | 74.79 (26.94) |
| Known Provider Continuity 3 yrs (std) | 44.79 (35.94) | 26.48 (32.12) | 49.84 (35.29) | 57.32 (31.55) | 41.92 (28.04) | 58.92 (31.46) |
| Known Provider Continuity 5 yrs (std) | 37.64 (34.36) | 21.62 (28.01) | 42.06 (34.64) | 49.57 (32.69) | 34.03 (27.46) | 51.18 (32.77) |
| Known Provider Continuity - Multiple Providers 1 yr (std) | 37.35 (38.66) | 12.20 (28.03) | 44.28 (38.30) | 52.08 (35.51) | 29.78 (36.37) | 54.38 (34.61) |
| Known Provider Continuity - Multiple Providers 3 yrs (std) | 36.77 (30.82) | 12.89 (22.17) | 43.35 (29.59) | 53.27 (25.21) | 34.92 (27.13) | 55.17 (24.23) |
| Known Provider Continuity - Multiple Providers 5 yrs (std) | 36.16 (28.34) | 13.48 (20.55) | 42.41 (26.97) | 53.18 (22.39) | 36.55 (24.32) | 54.89 (21.46) |
| % of visits with physician seen most 1 yr (std) | 48.93 (35.17) | 34.48 (39.68) | 52.91 (32.73) | 53.93 (29.03) | 48.87 (34.57) | 54.46 (28.35) |
| % of FP visits with FP seen most 1 yr (std) | 58.95 (43.62) | 30.06 (42.09) | 66.91 (40.57) | 71.73 (36.89) | 45.92 (43.14) | 74.40 (35.12) |
| % of FP visits with FP seen most 3 yrs (std) | 57.98 (33.66) | 30.08 (29.98) | 65.67 (30.40) | 73.28 (25.91) | 51.51 (29.58) | 75.53 (24.42) |
| % of FP visits with FP seen most 5 yrs (std) | 57.05 (30.81) | 30.76 (26.75) | 64.29 (27.76) | 73.05 (23.14) | 53.88 (25.71) | 75.04 (21.92) |
| Wolinsky 1 yr (std) | 36.21 (48.06) | 11.33 (31.70) | 43.06 (49.52) | 52.93 (49.91) | 27.19 (44.50) | 55.60 (49.69) |
| Wolinsky 3 yrs (std) | 35.67 (40.25) | 11.58 (25.28) | 42.31 (41.07) | 53.79 (40.44) | 30.82 (36.20) | 56.17 (40.11) |
| Wolinsky 5 yrs (std) | 35.12 (37.29) | 11.99 (23.23) | 41.50 (37.90) | 53.82 (36.78) | 33.19 (33.11) | 55.95 (36.48) |
| Modified Continuity Index (std) | 26.68 (25.61) | 12.12 (20.09) | 30.69 (25.51) | 35.03 (24.84) | 22.60 (24.02) | 36.32 (24.57) |
| Comorbidity: Charelson Score Mean (std) | 0.27 (1.09) | 0.07 (0.53) | 0.32 (1.19) | 0.40 (1.32) | 0.17 (0.87) | 0.42 (1.36) |

* Combination of the Statistics Canada community size (CSizeMIZ) variable for urban areas, and of the statistical area classification type (SACtype) variable for rural areas.

CAMCA: Census Metropolitan Area/Census Agglomeration

FP: Family Physician
