# Supplementary material for: Reporting a regular medical doctor index: A new measure of patient-physician affiliation for health administrative data
Source: PLoS One. 2024 Dec 2;19(12):e0314381. doi: 10.1371/journal.pone.0314381 (PMC11611086; doi:10.1371/journal.pone.0314381)
Supplement: S5 Table — Characteristics are reported for the full unweighted and weighted CCHS cohort. (DOCX) [file pone.0314381.s005.docx]

**S5 Table. Characteristics of Canadian Community Health Survey (CCHS) respondents reporting having a regular medical doctor and those who reported not having a regular medical doctor**. Characteristics are reported for the full unweighted and weighted CCHS cohort.

|  | Total full CCHS cohort | Report not having a regular medical doctor | Report having a regular medical doctor | Total full CCHS cohort  (weighted) | Report not having a regular medical doctor (weighted) | Report having a regular medical doctor  (weighted) |
| --- | --- | --- | --- | --- | --- | --- |
| N | N=60,968 | N=13,077 | N=47,891 | N=60,981.64 | N=15,812.54 | N= 45,169.10 |
| Age (SE) | 54.10 (0.08) | 37.47 (0.15) | 50.57 (0.09) | 44.63 (0.12) | 35.94 (0.20) | 47.68 (0.13) |
| Female | 54.08% (0.20) | 39.51 (0.43) | 58.06 (0.22) | .50.64 (0.34) | 37.62 (0.69) | 55.20 (0.39) |
| Neighbourhood Income Quintile N(%) | 3.03 (0.01) | 2.99 (0.01) | 3.04 (0.01) | 3.04 (0.01) | 2.96 (0.02) | 3.07 (0.01) |
| *Rurality (Census Size MIZ) | 3.42 (0.009) | 3.10 (0.02) | 3.51 (0.01) | 2.59 (0.01) | 2.22 (0.02) | 2.73 (0.02) |
| # of Ambulatory visits 1 yr (std) | 5.39 (0.04) | 2.26 (0.05) | 6.24 (0.05) | 5.13 (0.06) | 2.30 (0.07) | 6.13 (0.07) |
| # of Ambulatory visits 3 yr (std) | 5.23 (0.03) | 2.23 (0.04) | 6.05 (0.03) | 4.96 (0.04) | 2.25 (0.05) | 5.91 (0.05) |
| # of Ambulatory visits 5 yr (std) | 5.13 (0.03) | 2.26 (0.03) | 5.91 (0.03) | 4.85 (0.04) | 2.27 (0.05) | 5.76 (0.04) |
| # of FP visits 1 yr (std) | 3.00 (0.02) | 1.34 (0.02) | 3.46 (0.02) | 2.70 (0.02) | 1.30 (0.03) | 3.19 (0.03) |
| # of FP visits 3 yrs (std) | 2.98 (0.02) | 1.34 (0.02) | 3.43 (0.02) | 2.67 (0.02) | 1.30 (0.02) | 3.15 (0.02) |
| # of FP visits 5 yrs (std) | 2.96 (0.01) | 1.37 (0.02) | 3.40 (0.02) | 2.67 (0.02) | 1.31 (0.02) | 3.15 (0.02) |
| # of ED visits (std) | 0.43 (0.01) | 0.26 (0.01) | 0.48 (0.01) | 0.38 (0.01) | 0.25 (0.02) | 0.43 (0.01) |
| # of Weekend visits (std) | 0.60 (0.01) | 0.46 (0.01) | 0.64 (0.01) | 0.49 (0.01) | 0.36 (0.02) | 0.53 (0.01) |
| UPC Index Mean (std) | 48.66 (0.20) | 23.63 (0.37) | 55.49 (0.23) | 45.84 (0.34) | 24.94 (0.64) | 53.16 (0.39) |
| Known Provider Continuity 1 yr (std) | 56.62 (0.15) | 38.49 (0.35) | 65.40 (0.16) | 56.71 (0.27) | 37.02 (0.55) | 63.60 (0.28) |
| Known Provider Continuity 3 yrs (std) | 44.89 (0.15) | 26.55 (0.28) | 49.87 (0.16) | 42.16 (0.24) | 25.17 (0.44) | 48.11 (0.27) |
| Known Provider Continuity 5 yrs (std) | 37.71 (0.14) | 21.65 (0.25) | 42.09 (0.16) | 34.90 (0.22) | 20.28 (0.38) | 40.02 (0.26) |
| Known Provider Continuity - Multiple Providers 1 yr (std) | 37.49 (0.16) | 12.27 (0.25) | 44.38 (0.17) | 34.37 (0.26) | 11.38 (0.36) | 42.42 (0.30) |
| Known Provider Continuity - Multiple Providers 3 yrs (std) | 36.91 (0.12) | 12.97 (0.19) | 43.44 (0.14) | 33.81 (0.20) | 11.96 (0.29) | 41.47 (0.23) |
| Known Provider Continuity - Multiple Providers 5 yrs (std) | 36.29 (0.11) | 13.54 (0.18) | 42.50 (0.12) | 33.22 (0.19) | 12.41 (0.27) | 40.51 (0.20) |
| % of visits with physician seen most(std) | 48.94 (0.14) | 34.52 (0.35) | 52.87 (0.15) | 47.52 (0.25) | 34.51 (0.56) | 52.08 (0.26) |
| % of FP visits with FP seen most 1 yr (std) | 59.07 (0.18) | 30.18 (0.37) | 66.96 (0.19) | 57.00 (0.30) | 32.70 (0.62) | 65.51 (0.32) |
| % of FP visits with FP seen most 3 yrs (std) | 58.09 (0.14) | 30.15 (0.26) | 65.72 (0.14) | 55.76 (0.22) | 31.94 (0.44) | 64.09 (0.23) |
| % of FP visits with FP seen most 5 yrs (std) | 57.14 (0.12) | 30.80 (0.23) | 64.34 (0.13) | 54.64 (0.20) | 32.18 (0.37) | 62.51 (0.21) |
| Wolinsky 1 yr (std) | 36.45 (0.19) | 11.45 (0.28) | 42.56 (0.19) | 32.58 (0.26) | 10.63 (0.31) | 40.27 (0.30) |
| Wolinsky 3 yrs (std) | 35.94 (0.16) | 11.73 (0.22) | 43.29 (0.22) | 33.86 (0.31) | 10.33 (0.41) | 40.75 (0.37) |
| Wolinsky 5 yrs (std) | 35.38 (0.15) | 12.11 (0.20) | 41.74 (0.17) | 31.99 (0.23) | 12.21 (0.29) | 39.27 (0.27) |
| Modified Continuity Index (std) | 26.79 (0.10) | 12.14 (0.18) | 30.79 (0.12) | 24.85 (0.17) | 11.66 (0.26) | 29.47 (0.19) |
| Comorbidity: # of Charelson Score Mean (std) | 0.27 (0.004) | 0.069 (0.005) | 0.33 (0.005) | 0.22 (0.006) | 0.06 (0.006) | 0.28 (0.007) |

* Combination of the Statistics Canada community size (CSizeMIZ) variable for urban areas, and of the statistical area classification type (SACtype) variable for rural areas.

FP: Family Physician
